# Molecular links between endocrine, nervous and immune system during chronic stress

**DOI:** 10.1002/brb3.1960

**Published:** 2020-12-08

**Authors:** Roberto Zefferino, Sante Di Gioia, Massimo Conese

**Affiliations:** ^1^ Department of Medical and Surgical Sciences University of Foggia Foggia Italy

**Keywords:** circadian rhythm, cortisol, interleukin‐1β, melatonin, sickness behavior

## Abstract

**Introduction:**

The stress response is different in various individuals, however, the mechanisms that could explain these distinct effects are not well known and the molecular correlates have been considered one at the time. Particular harmful conditions occur if the subject, instead to cope the stressful events, succumb to them, in this case, a cascade reaction happens that through different signaling causes a specific reaction named “sickness behaviour.” The aim of this article is to review the complex relations among important molecules belonging to Central nervous system (CNS), immune system (IS), and endocrine system (ES) during the chronic stress response.

**Methods:**

After having verified the state of art concerning the function of cortisol, norepinephrine (NE), interleukin (IL)‐1β and melatonin, we describe as they work together.

**Results:**

We propose a speculative hypothesis concerning the complex interplay of these signaling molecules during chronic stress, highlighting the role of IL‐1β as main biomarker of this effects, indeed, during chronic stress its increment transforms this inflammatory signal into a nervous signal (NE), in turn, this uses the ES (melatonin and cortisol) to counterbalance again IL‐1β. During cortisol resistance, a vicious loop occurs that increments all mediators, unbalancing IS, ES, and CNS networks. This IL‐1β increase would occur above all when the individual succumbs to stressful events, showing the Sickness Behaviour Symptoms. IL‐1β might, through melatonin and *vice versa*, determine sleep disorders too.

**Conclusion:**

The molecular links here outlined could explain how stress plays a role in etiopathogenesis of several diseases through this complex interplay.

## Graphical abstract

Central nervous system (CNS), immune system (IS), and endocrine system (ES) operate together during the chronic stress response. We propose that this complex molecular network is altered in sickness behaviors.

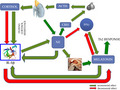

## 1 INTRODUCTION

The first observations about stress were made by Selye, who can be considered the “father” of stress. He described stress as general adaption syndrome (GAS) and specified the role of hypothalamic‐pituitary‐adrenocortical axis (HPA) and sympathetic‐adrenal‐medullary (SAM) system. He identified three phases and already underlined that the two first phases, alarm reaction and resistance, could not appear as dangerous for the health, while the third phase that he named exhaustion had to be considered differently, potentially hazardous (Selye, 1956). In fact, recurring stress appears to be dangerous, because it exceeds the ability to cope with it. Considering only the HPA, we can notice that in acute stress cortisol first is incremented and then decreases, instead during chronic stress cortisol lacks its circadian rhythm. This leads to the glucocorticoid resistance where the increment of cortisol associates with a lack of effect. Moreover, during the stress, if the effects of different hormones and signaling paracrine molecules have been studied thoroughly, their interplay was not considered by many. Recent attention has focused on the stress causes considering subjective and objective aspects regarded as both important. For example, work‐related stress can derive either by objective aspects (e.g., increased job demand) or by neutral situations that the worker perceives as stressful. In this respect, it might be useful reporting the stress definition made by S. Cohen: “*The experience of negative events or the perceptions of distress and negative affect that are associated with the inability to cope with them*” (Cohen et al., 2001). This sentence explains very well, in our opinion, that not only the perception can realize an important difference explaining distinct responses to the events but also the individual response to the stressors, the way how individual copes the stressors can elucidate the lack of uniformity in reaction to the same event. According to this, McEwen and his team have recognized that protective and damaging effects of the biologic response to stressors should be named allostasis and allostatic overload, respectively (McEwen, 1998). In particular, allostasis is distinguished from homeostasis in that this it is an adaptive process that tries to maintain homeostasis by promoting the release of glucocorticoids, catecholamines, and cytokines. On the other hand, allostatic overload refers to the response to prolonged stress, mediated by many neuroendocrine mediators (McEwen, 2008). Finally, neural mechanisms influence how an individual copes with this situation determining either vulnerability or resilience (Charney, 2004; Hodes et al., 2015).

The aim of this article is to highlight the links between central nervous system (CNS) and immune system (IS) considering also the role of endocrine system (ES) and circadian rhythm (CR). In this regard, it might be useful to study the stress evaluating the complex link between cortisol, interleukin‐1β (IL‐1β), norepinephrine (NE), and melatonin during chronic stress. Since IL‐1β can be considered as a bidirectional mediator between CNS and IS; then, we remark as CNS might act on IS and ES and as they might interfere between themselves. Although the stress response has been thoroughly studied at the molecular level, many studies considered one molecule at the time. Our article would like to show the complex molecular interplay that could explain the various effects of stressful events on different subjects, considering as everyone's response depends on various circumstances. Then, we hypothesize that IS through IL‐1β, ES using cortisol, CNS considering the role of NE, together may interfere on melatonin secretion modifying the CR. In the network between CNS, IS, and ES, an important role is played by melatonin because it exerts effects on above considered systems also through CR. Moreover, it is known the role of melatonin on sleep and as this latter is critical for wellness and mental and physical health too. Many observations in these different fields were reported, but nobody explored the links between different molecules that regulate particular systems capable to control themselves through positive or negative feedbacks. The choice to light up as the circumstances and events that subjects meet in their life may deteriorate the wellness or mental health and worsen the physical health interfering on the above‐indicated systems is a fascinating challenge that we would like to take up: It might be an outlook for future research. Particularly interesting appears the role of IL‐1β, since this cytokine acts also as sleep regulatory substance and it is known as the sleep is essential for wellness and mental and physical health and as the same sleep buffers the IS maintaining the right balance between Th1 and Th2 response.

## 2 SEARCH STRATEGY

The following search items, combined with the Boolean term “AND,” were used to perform an electronic search in the PubMed, EMBASE, and Scopus databases: chronic stress, endocrine system, immune system, central nervous system, cortisol, melatonin, norepinephrine, cytokines, interleukin‐1, interferon, hypothalamic‐pituitary‐adrenal axis, hypothalamic‐pituitary‐gonadal axis, circadian rhythm, major depressive disorder, sickness behaviors, and sleep regulatory substance. This is a narrative review of the literature and not a systematic review.

## 3 GLUCOCORTICOIDS AND STRESS

**FIGURE 1:**
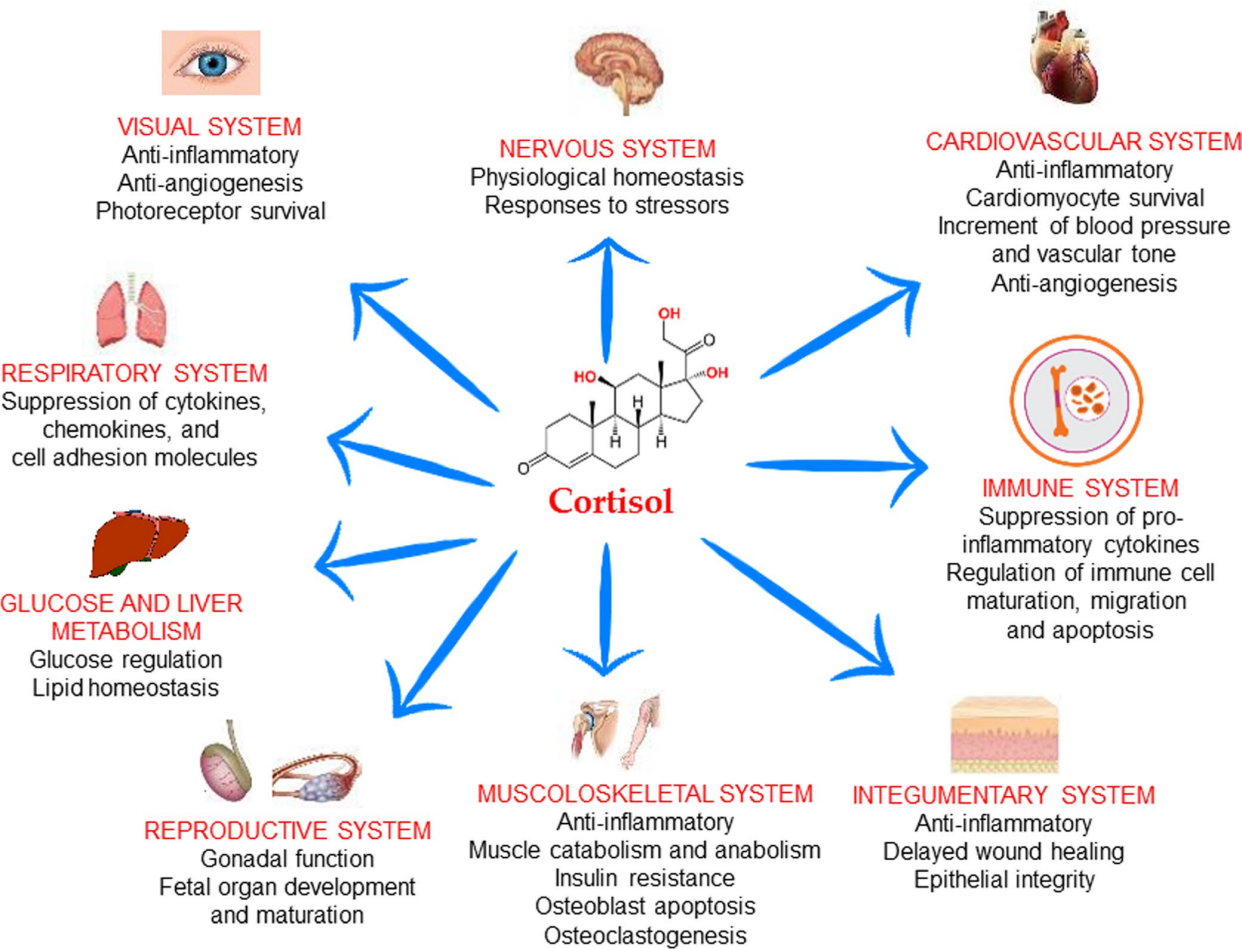
Role of cortisol in health. This schematic represents the roles of glucocorticoids (cortisol) in major organ systems of the human body. Glucocorticoids act through genomic and nongenomic actions (see text for details)

Cortisol was named “stress hormone” because it augments in alarm reaction; however, a low increment of this hormone is not dangerous for health in that cortisol regulates numerous organ functions in the body (Figure 1). Cortisol has a distinct circadian rhythm, regulated by the central pacemaker localized in the suprachiasmatic nucleus, which activates the hypothalamic‐pituitary‐adrenal (HPA) axis. Cortisol levels are at the lowest levels at around midnight, start to decrease at 02:00–03:00 and peak in the morning, declining back to nadir throughout the day (Debono et al., 2009). The lack of this rhythm may result in many diseases. In fact, we may observe it in adrenal insufficiency (Chan et al., 2010) but the lack of this rhythm might even lead to cancer (Sulli et al., 2019). Mortality in patients with breast cancer is associated with flattened cortisol rhythms (Sephton et al., 2000), and similarly patients with colorectal cancer and higher mortality cancer show erratic periods of rest/activity and poor sleep (Innominato et al., 2018; Mormont et al., 2000).

Besides by the HPA axis, two isoforms of the enzyme 11β‐hydroxysteroid dehydrogenase (11β‐HSD) regulate cortisol levels: 11β‐HSD1 is bidirectional but it is believed to act in vivo generating predominantly the active cortisol from the inactive cortisone, while, in contrast 11β‐HSD2 acts to convert cortisol to cortisone (Tomlinson et al., 2004).

The genetic actions of glucocorticoids are well known (Argentieri et al., 2017). When bound to glucocorticoids (GCs), the glucocorticoid receptor (GR), resident in the cytoplasm, translocates to the nucleus and modifies the synthesis of many metabolic, immune, and inflammatory proteins through either transactivation or transrepression. The first is operated by direct binding to glucocorticoid response elements (GREs) and leads an up‐regulation of immune‐ and metabolic‐related proteins, while the second acts via the influence on the activity of transcription factors without contacting directly DNA and has as a result the down‐regulation of proinflammatory and immunosuppressive proteins. Hormone binding and nuclear translocation of the GR is negatively regulated by the immunophilin FK506‐binding proteins (FKBP) 51 (Wochnik et al., 2005). FKBP expression itself is induced by GCs as part of an intracellular ultra‐short negative feedback loop for GR activity (Vermeer et al., 2003). Interestingly, polymorphisms of the *FKBP5* gene are associated with increasing activity of the protein, causing GR resistance (Binder, 2009).

Glucocorticoids also exert nongenomic actions by activating transduction pathways and interacting with cellular membranes (Kadmiel et al., 2013; Strehl et al., 2013). Genomic and nongenomic mechanisms of action of GCs makes these hormones a key regulator of many fundamental physiological systems, among which the immune, cardiovascular, and nervous ones have the pre‐eminence (Kadmiel et al., 2013; Kalsbeek et al., 2012; Smith et al., 2006).

Conceivably, a resilient phenotype can be conferred by the hyperactivation of the HPA axis, through a mechanism called stress inoculation. If GC administration confers a proresilience status is not well known, likely involving traumatic memory consolidation such as in the posttraumatic stress disorder (Kearns et al., 2012).

In the opinion of several authors, vulnerability and resilience depend on individual differences that are able to induce different neuroimmune and neuroendocrine responses. (Charney, 2004; Hodes et al., 2015). If the individual is overactive the stress vulnerability increases due to unresolved stress responses and could induce mood disorders (Charney, 2004), although most individuals using appropriate coping strategies show resilience in the face of stress (Pfau et al., 2015; Russo et al., 2012).

Finally, it has to be remarked that HPA axis hyperactivity and consequent GC resistance might represent the link between chronic stress and major depressive disorder (MDD), diabetes, and metabolic syndrome (Brown et al., 2004; Menard et al., 2017).

## 4 STRESS, GLUCOCORTICOIDS, AND IMMUNE SYSTEM

It is known that inflammatory activity is controlled by different CNS processes (Slavich et al., 2014). Through this control, CNS can prepare body to the injury before that infection occurs. By redistributing and trafficking of innate immune cells, the body anticipates the response to pathogen. It was also demonstrated that immune system could response if an individual is exposed to social conflict, evaluation, rejection, or exclusion especially if these conditions appear as dangerous. However, this ancestral host defense mechanism is able to increase the risk for viral infection and inflammation‐related disease. Sympathetic nervous system (SNS) and HPA axis would act together by releasing NE. In particular, it has been proposed (Cole et al., 1998; Lee et al., 2000) that NE would be capable to supress transcription of antiviral type I interferon (IFN) genes and up‐regulate transcription of the proinflammatory immune response genes IL‐1, TNF, and IL‐6. This could lead to increments in systemic inflammatory activity (Cole et al., 2010; Grebe et al., 2010). In particular, IL‐1β is a potent proinflammatory cytokine, playing important roles a part as pyrogen. It induces prostaglandin synthesis, neutrophil and T‐ and B‐cell activation and antibody production, as well as favors fibroblast proliferation and collagen production. It appears useful to remember its synergism with IL‐12 inducing IFN‐γ synthesis from Th1 cells following cell stimulation with IL‐12 (Tominaga et al., 2000).

The HPA axis plays an important role in the control of inflammation, that is, innate immunity, through cortisol (Berkenbosch et al., 1987; Besedovsky et al., 1986; Sapolsky et al., 1987). Indeed, GCs are potent anti‐inflammatory agents and act by inducing apoptosis in monocytes, macrophages, and T cells (Amsterdam et al., 2002) and suppressing the NF‐κB pathway (De Bosscher et al., 2003).

This control on inflammation occurs in basal condition, whereas under other circumstances a different set of mechanisms can emerge, leading to HPA axis‐related increase in inflammation (Avitsur et al., 2001; Miller et al., 2002). This process was named as *glucocorticoid resistance*, whereby it appears that immune cells become less sensitive to the effects of glucocorticoids (Schleimer, 1993). When glucocorticoid resistance develops, “fight or flight” responses to social threat are altered and determines exaggerated inflammation, particularly if these responses occur frequently. Thus, GC resistance provoked by chronic stress may determine a reduction in anti‐inflammatory and proresolving actions of GCs and a prolonged inflammatory process (Cohen et al., 2012). Different authors retain that these mechanisms could affect mental and physical health (Marques et al., 2009; McEwen, 1998, 2008; McEwen et al., 1999). For example, subjects with MDD have flatter diurnal slopes than persons without MDD, and glucocorticoid sensitivity can in part explain these higher overall cortisol concentrations (Anacker et al., 2011; Fries et al., 2005; Jarcho et al., 2013; Pace et al., 2007, 2011).

Cortisol exert important effects on adaptive IS. This was investigated by Elenkov and Chrousos (Elenkov et al., 2002), which verified that cortisol was able to polarize naive CD4 + T cells toward the T helper (Th)2 subset. This polarization would make the subject more susceptible to infective disorders and autoimmune diseases, as well as also less reactive toward cancer.

In this context, it is important to mention Palumbo et al. (Palumbo et al., 2010), who showed that BALB/c mice were less protected by the stress than C57BL/6 mice, this correlating with a differential regulation of the Th1/Th2 cytokine balance. In fact, stress induces a Th1 response in C57BL/6 mice with an increase of IFN production that could protect against the neurodegenerative processes. Instead in BALB/c mice an increase in Th2 cytokines and a decrease in IFN correlate with poor memory performances during chronic mild stress. Thus, it might happen that during glucocorticoid resistance state, cortisol polarizes IS toward a Th2 response (Elenkov et al., 2002),

## 5 NEUROINFLAMMATION AND STRESS

Different authors retain that stress is able to modify inflammatory events in CNS and in immune system, such responses promoting behavioral vulnerability and resilience. It is known that the stress can induce monocytosis (Ginhoux et al., 2014). Moreover, the acute stress induces an adaptive response, but chronic stress promote sustained, unresolved inflammation, and leukocytosis, these last considered hallmark symptoms of depression (Maes et al., 1992). In animal models of depression‐like behavior increased proinflammatory cytokines levels were reported (Grippo et al., 2005; Hodes et al., 2014). Different research groups showed that IL‐1β tumor necrosis factor (TNF), or lipopolysaccharide (LPS) were able to promote proinflammatory genes and proteins in the brain (van Dam et al., 1992; Laye et al., 1994; Quan et al., 1999). Moreover, they induce, in rodents, sickness behaviors characterized by social withdrawal, loss of appetite, decreased motor activity, and cognitive deficits (Dantzer et al., 2008). More recently, a similar correlation between immune response and sickness behavior was found in zebrafish. It was observed that zebrafish inoculated intraperitoneally with *Aeromonas hydrophila* bacterin had not only a systemic inflammatory response that altered the expression of cytokines gene in the brain but also alterations in behavioral parameters (Kirsten et al., 2018). Overall, these results show that even though specific behavior varies from species to species, the sickness behavior seems to be conserved among all vertebrates.

Interestingly, Hodes and colleagues observed that mice that showed increment of IL‐1β and IL‐6 in the blood after a single exposure to an aggressor became susceptible, instead mice resilient had not this increase (Hodes et al., 2014).

Some authors (Banks et al., 1994, 1995; Hodes et al., 2015) reported that cytokines are able to cross Blood‐Brain Barrier (BBB), so they can act on astrocytes, neurons, and microglia. Brain endothelial cells play an important role in the inflammatory response underlying chronic stress because they are capable to produce and secrete cytokines (Verma et al., 2006).

Two independent research groups demonstrated that rodents vulnerable to stress following LPS stimulation had an important increment of IL‐1β, IL‐6, and TNF‐α, while unstressed controls showed no increase (Frank et al., 2007; Wohleb et al., 2011). Following studies confirming such results, (Koo et al., 2008) reported that inhibition of IL‐1β receptor rescues anhedonia in rats exposed to chronic stress (Koo et al., 2008). Maier et al (Maier et al., 1995) showed that such receptor blockage prevented failure to escape in the Learn Helplessness paradigm, confirming the role of IL‐1β in stress vulnerability. Mason et al. described an interesting association between diabetes, atherosclerosis, myocardial infarction, and rheumatoid arthritis and MDD. Patients with this comorbidity also tend to exhibit enhanced activation of the NLRP3 inflammasome complex (Mason et al., 2012).

**FIGURE 2:**
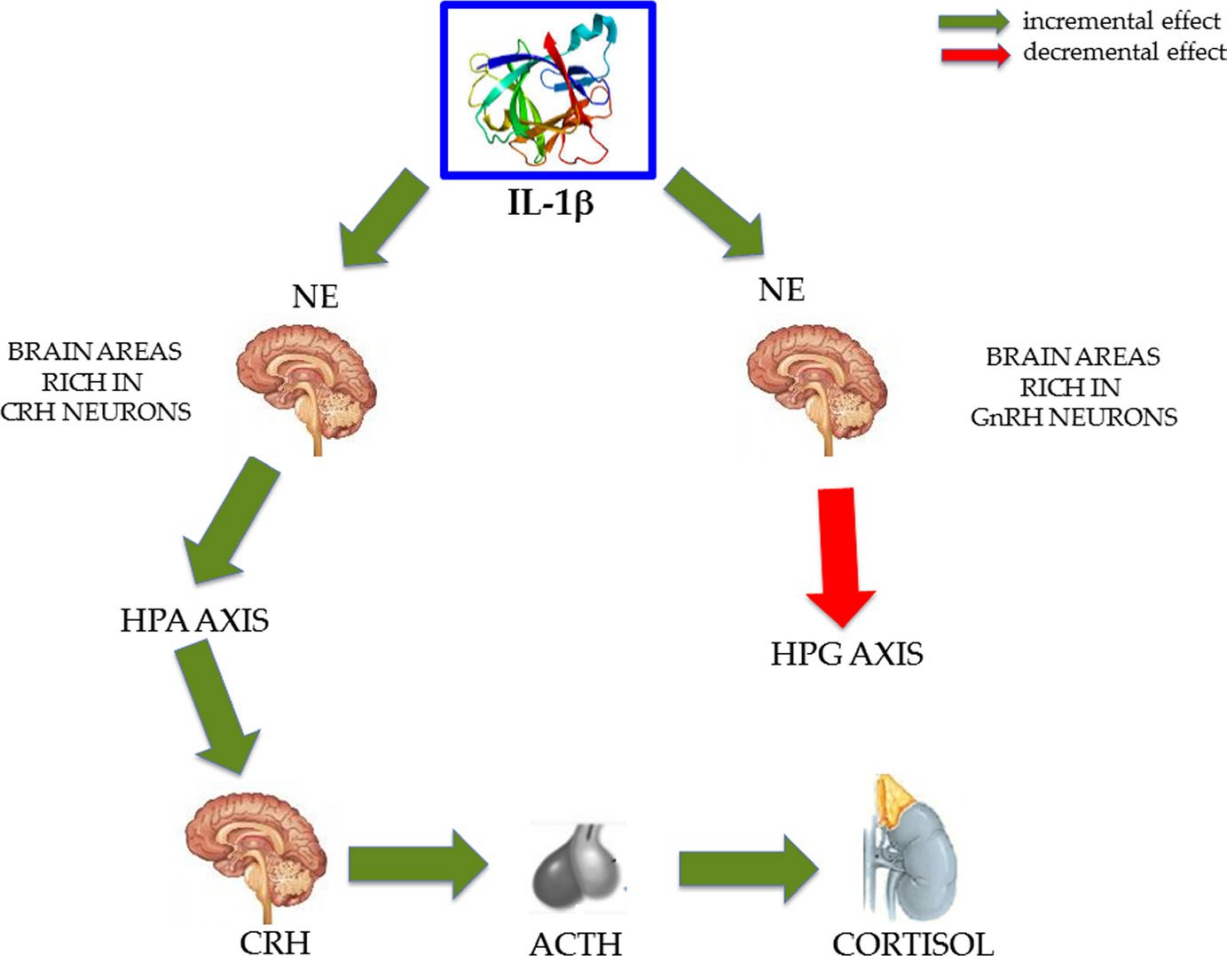
The role of Il‐1β in the endocrine system. IL‐1β, produced by microglia, can activate the hypothalamic‐pituitary‐adrenocortical (HPA) axis and suppress the hypothalamic‐pituitary‐gonadal (HPG) axis, by increasing or decreasing noradrenaline (NE) in specific brain regions enriched in corticotrophin‐releasing hormone (CRH) neurons and gonadotropin‐releasing hormone (GnRH), respectively. The protein structure of IL‐1β was taken from the RCSB‐protein data bank (www.rcsb.org/)

In addition to the circumstance that activated macrophages as well as microglia are able to produce IL‐1β, it was demonstrated its ability to activate the HPA axis and suppress the hypothalamic‐pituitary‐gonadal (HPG) axis (Berkenbosch et al., 1987; Sapolsky et al., 1987; Sirivelu et al., 2012). For this reason, O'Connor and colleagues, underlining these neuroendocrine effects, proposed its role in the homeostatic adaptation during an immune challenge (O'Connor et al., 2000). These effects are produced by IL‐1β acting on specific brain regions regulating HPA and HPG axes (Figure 2). These brain areas rich in corticotrophin‐releasing hormone (CRH) neurons are responsive to immune stimulation and regulate HPA or stress axis and constitute the link between IS and CNS (Herman et al., 2003). Gonadotropin release hormone (GnRH) neurons and CRH neurons are stimulated both by NE (Kadmiel et al., 2013). Thus, IL1β activates the HPA axis by stimulating CRH neurons, the result being an increment of adrenocorticotropin (ACTH) from the pituitary, and ultimately an increase in corticosterone secretion from the adrenal gland (Berkenbosch et al., 1987; Besedovsky et al., 1986; Brady et al., 1994; Sapolsky et al., 1987). However, it is unclear as IL‐1β incites CRH secretion, perhaps an increase of NE levels in the paraventricular nucleus (PNV) mediates this effect (DeKeyser et al., 2000; Schmidt et al., 2001). On this line, Sirilevu and colleagues showed in mice that NE in the PNV could be considered a mediator of the stress response induced by IL‐1β (Sirivelu et al., 2012).

Goshen and Yirmiya (Goshen et al., 2009) considered IL‐1 in stress response and suggested that either glia cells and neurons are capable to produce IL‐1 or that IL‐1 produced peripherally enters into the brain (Dinarello, 1996; Maier et al., 1998). IL‐1β produces its effect in the brain either by crossing the BBB (Banks et al., 1989) and by activation of vagal afferent fibers. This last circumstance is proved by vagotomy that was capable to block centrally mediated effects of peripheral immune activation (Gaykema et al., 2000; Watkins et al., 1995) and attenuate the effects of peripherally administered IL‐1 on the behavior (Bluthe et al., 1996). Moreover, Goshen and Yirmiya (Goshen et al., 2009) have hypothesized that immunological and psychological stress responses activate microglia, which produces de novo IL‐1 or secrete a prestored pool. The stress would induce stimulation of NE secretion within the brain and could stimulate this response. It is noteworthy that either IS and psychological stressors share many IL‐1 mediated effects including fever, alterations in peripheral immune parameters, neuroendocrine modulation, and sickness behavior symptoms. In addition, it has been demonstrated that there exists a two‐way relationship between brain IL‐1 and noradrenergic systems as the effects of IL‐1 on the hypothalamic noradrenergic neurotransmission are influenced by the activity of IL‐1 on HPA axis. As a result, many neurobehavioral mechanisms are modified by the stress‐induced IL‐1 mediated release of glucocorticoids. In particular, IL‐1 plays a key role in stress‐induced modulation of the process of memory functioning (Goshen et al., 2007).

Summarizing, it was assumed that whereas in some physiological condition low amounts of IL‐1 encourage the adaptive stress responses required for adequate coping, in severe and long‐term stress situations IL‐1 moderated various harmful cognitive and emotional effects of stress.

Krueger (Krueger, 2008) also suggested IL‐1β as a sleep regulatory substance (SRS), specifically it causes nonrapid eye movement sleep (NREMS), together with TNF, growth hormone‐releasing hormone (GHRH), adenosine, prostaglandin D2, that all produce the same results. Situations which promote internal production of IL‐1 or TNF, for example, unrestricted food intake (Hansen et al., 1998) or infections (Toth et al., 1988), stimulate NREMS (Krueger et al., 2007; Obal et al., 2003). That cytokines are implied in the physiological sleep supervision, and their relations to other SRSs have been described in many reviews (Kapsimalis et al., 2005; Krueger et al., 2007; Obal et al., 2003). Many laboratories have produced what is now enormous evidence concerning sleep deprivation‐enhanced IL‐1β, and the associated cytokine TNF, to symptoms connected to sleep privation, such as sensitivity to firing (Yi et al., 2004) and pain stimuli (Honore et al., 2006; Kawasaki et al., 2008; Kundermann et al., 2008), cognitive (Baune et al., 2008; Gambino et al., 2007; Trompet et al., 2008), memory (Banks et al., 2007; Dantzer, 2004; Pickering et al., 2007), and performance impairments (Banks et al., 2007), depression (Anisman et al., 2003; Vollmer‐Conna et al., 2004), sleepiness (Krueger et al., 2007; Moldofsky, 1995; Tringali et al., 2000), and fatigue (Anisman et al., 2003; Carmichael et al., 2006; Omdal et al., 2005). In addition, long‐term sleep deprivation is linked to pathologies as metabolic syndrome, (Hristova et al., 2006; Jager et al., 2007; Larsen et al., 2007) chronic inflammation (Frey et al., 2007; Hu et al., 2003), and cardiovascular disease (Yndestad et al., 2007). All of these sleep loss‐connected symptoms can be triggered by inoculation of exogenous IL‐1 and or TNF (Krueger et al., 2007; Obal et al., 2003), or in some cases stopped if these cytokines are suppressed (Depino et al., 2004; Larsen et al., 2007; Obal et al., 2003; Opp & Krueger, 1991).

Brain levels of IL‐1 mRNA and plasma levels of IL‐1 change along with sleep‐wake cycle with highest levels related to high sleep tendency (Fang et al., 1997, 1998). Antisomnogenic cytokines behave, in part, by suppressing production of prosomnogenic mediators. For example, IL‐10 stops IL‐1 and TNF secretion and rises the production of sleep‐inhibitory substances as CHR. IL‐1 and TNF may also constitute a connection between the circadian rhythm and sleep homeostasis. There are nictemeral rhythms in brain cytokines including IL‐1 and TNF (Krueger et al., 2007).

To conclude, IL‐1 is a cytokine which promotes neuroinflammation and is synthetized and secreted during innate immune reactions. Its production increases also in psychiatric disorders as depression and anxiety, making IL‐1 a biomarker of stress and sleep disorders. It is noteworthy to deepen the study of this cytokine because it appears linked to cortisol, epinephrine, and melatonin. These molecules control numerous pathway and systems (CNS, IS, and ES) these permit them to exert a mutual control. These molecular links and their characteristic functions could indicate new starting points in order to propose future researches to understand as different subjects do not show the same manifestations and the same prognosis even if they have the same disease, verifying as the stress can interfere on progress and onset of the diseases, including those of infective origin. Moreover, the role of this cytokine in the sleep deprivation could explain as the stress can act worsening mental health.

## 6 MELATONIN

Central nervous system, IS, and ES network use NE, IL‐1β and cortisol, nevertheless melatonin appears as a molecule capable to play different roles either in both IS and ES. Its secretion and its function are controlled by NE and IL‐1β. Moreover, if in the past it was considered only as a hormone, today its role is increasingly investigated as a multifunctional molecule, and because of this, we would like to consider its role in chronic stress.

Melatonin has been revealed and studied from bovine pineal gland by the dermatologist Aaron Lerner in the 1958 (Claustrat et al., 2005). It is the major hormone produced by the pineal gland. Other sources are retina, gut, skin, platelets, and the bone marrow (Liu et al., 2004; Stefulj et al., 2001). Melatonin has indole form (N‐acetyl‐5‐methoxytryptamine) and is produced from serotonin. In spite of the fact that melatonin has been widely found in the animal world, it was also observed in higher plants and bacteria. It is probable that melatonin is one of early compounds which was present on earth to arrange some basic events of life.

The principal physiological tasks of melatonin are associated with hormonal properties, even if it may also present autocrine or paracrine characteristics, for example, in the retina or the gut (Tan et al., 2003). The pineal gland was initially considered to be a working neuroendocrine transformer of environmental data in animals, especially in photoperiodic animals. For numerous years, the information had been extrapolated to humans. Today, partial knowledge of the role of melatonin in human physiology and pathology has arisen, but many functions and outcomes of melatonin remain in the dark.

Melatonin shows high lipid and water solubility (octanol/water coefficient of partition = 13) which makes the passing across cell membranes easier (Pardridge et al., 1980). Because there is no storage room for melatonin, the plasma hormone figure testifies precisely the pineal activity (Reiter, 1991).

The secretion takes place at night, with highest plasma levels around 03:00–04:00 am, changing with chronotype, while daytime levels are untraceable, or low in people at rest. This nyctohemeral cycle shows the most pronounced extent observed for a hormone, even more pronounced than that of cortisol. Still, it is very replicable from day to day in the same person and illustrates one of the firmest circadian rhythms. It offers a nice assessment of the secretion of melatonin, in the lack of renal or hepatic anomaly (Grof et al., 1985). The light/dark rhythm is the main Zeitgeber of the control system of melatonin secretion. The melatonin cycle is started around the dark period. The photic data are transferred to the central pacemaker through the retino‐hypothalamic fibers: over the day, in the presence of light, the output from the retino‐hypothalamic tract suppresses the synthesis of melatonin. Artificial room light of adequate intensity and duration applied at night suppresses melatonin secretion (Lewy et al., 1980). Moreover, after light exposition for various sequential nights, the melatonin secretion eludes the inhibitory effect and gradually shifts to the morning. The neural pathway of the SNC from the hypothalamus to the pineal gland runs first through the superior part of the cervical spinal cord, where synaptic connections are made up with preganglionic cell bodies of the superior cervical ganglia (SCG) of the sympathetic chains. Then, neural cells in the SCG dispatch projections to the pineal gland. The major neurotransmitter governing the pineal gland is norepinephrine, which is liberated at night, in reply to exciting signals which originate in the SNC. β1‐adrenergic blockers inhibit the nocturnal melatonin secretion (Cagnacci, 1996).

Current acquired evidence indicates that the pineal gland can play a key role in adjusting the immune response (Guerrero et al., 2002). Moreover, the relationship between the pineal gland and the immune system is two‐sided since interleukins and cytokines (such as IFN‐γ) influence melatonin synthesis and release (Hardeland et al., 1999). IFN‐γ increased the melatonin making by the pineal gland cells in vitro (Withyachumnarnkul et al., 1990), rather dosing of recombinant IL‐1β suppressed serum melatonin levels in rats via a receptor process (Mucha et al., 1994), and TNF‐α generated by the pineal gland microglia inhibited the synthesis of melatonin (da Silveira Cruz‐Machado et al., 2012).

Also the immune defense system is a resource of extrapineal melatonin, particularly peripheral blood mononuclear cells, circulating leucocytes, and macrophages are able to produce melatonin. On the other side, elevated concentrations of melatonin incremented IL‐1β levels in mice splenocytes (Arias et al., 2003). Experimental tests on trauma‐hemorrage in mice led to an immunosuppressed condition with low rates of IL‐1 and IL‐6 production, which were rebuilt to basal control ranges after therapy with melatonin (Wichmann et al., 1996). Contrarily, melatonin shows different attitude in conditions with worsened immune responses. Melatonin lowers neutrophil permeation and levels of the mediators of inflammation during rat heartstroke‐provoked lung inflammation and airway hyperreactivity (Chen et al., 2011; Lin et al., 2011).

A two‐faced impact of melatonin on the phorbol myristate acid (PMA)‐induced respiratory burst in human neutrophils has also been characterized; while low rates (10 nM) improve the response, high rates (2 mM) suppressed it (Pieri et al., 1998). Moreover, melatonin disabled the increased production of proinflammatory mediators, above all cytokines, in a great number of in vivo models of inflammation (Agil et al., 2013; Ara et al., 2011; Chahbouni et al., 2010; Chen et al., 2011; Esposito et al., 2008; Ganguly et al., 2012; Gitto et al., 2004, 2012; Gulben et al., 2010; Jang et al., 2013; Jung et al., 2009, 2010; Kang et al., 2011; Kara et al., 2013; Kaur et al., 2013; Kireev et al., 2008; Kunak et al., 2012; Li et al., 2005; Lin et al., 2011; Mazzon et al., 2006; Mei et al., 2002; Negi et al., 2011; Ochoa et al., 2011; Olcese et al., 2009; Ozen et al., 2007; Sener et al., 2006; Tahan et al., 2011; Tsai et al., 2011; Tyagi et al., 2010; Veneroso et al., 2009; Wang et al., 2005; Xu et al., 2007; Yang et al., 2011; Yip et al., 2013).

Early in vitro studies proposed that melatonin elicits the Th1 arm of the adaptive IS (Garcia‐Maurino et al., 1999). Substimulated peripheral blood mononuclear cells showed increased production of Th1 cytokines, like IFN‐γ and IL‐2, after in vitro melatonin supplement (Garcia‐Maurino et al., 1997, 1999). The daytime rhythmicity of human cytokine secretion showed that the IFN‐γ/IL‐10 peak takes place during the early morning, this climax definitely related to plasma melatonin (Petrovsky et al., 1997), implying a melatonin/Th1 causal link. On the contrary, melatonin substantially decreased the splenic CD19^+^ B‐cell population in mice with membranous nephropathy and reduced the TNF‐α, IL‐1β and IFN‐γ overexpression (Wu et al., 2012). Additional in vivo studies have displayed the ability of melatonin to stimulate a Th2 response in different models. It was shown first that high doses of melatonin promoted the production of the Th2 cytokine IL‐4 in bone marrow lymphocytes (Maestroni, 1995). Early overnight sleep caused a shift in the Th1/Th2 cytokine balance toward higher Th1 activity, while the Th2 response was dominant during a late sleep. A firm decline in TNF‐α‐producing CD8^+^ cells was also detected during sleep (Dimitrov et al., 2004), proposing a correlation between melatonin and the Th2 response. Similarly, the lack of melatonin due to pinealectomy polarized rat thymic Th1/Th2 cells toward a Th1 response by raising the production of IFN‐γ and reducing IL‐10 levels, suggesting that melatonin bends the immune response toward Th2 supremacy (Kelestimur et al., 2006). Growing doses of melatonin (0.25–1 mg/kg) given to mice infected with Venezuelan equine encephalomyelitis virus (VEEV) substantially rised serum levels of TNF‐α, IL‐1β and IFN‐γ. Blockage of IL‐1β with antimurine IL‐1β antibodies entirely canceled the protective role of melatonin, implying that that IL‐1β is the main target for the fast viral clearance caused by melatonin (Valero et al., 2002).

Therefore, melatonin has not to be considered alone, but it needs to be deemed together with IL‐1β, cortisol, and NE. Particularly, these molecules act as a network where they control each other determining and influencing stress response and conversely adjustments in ES, IS, and CNS. The study of manner how they act together is difficult and we can only speculate without hiding that such hypothesis will have to be carefully verified through future research.

## 7 CONCLUDING REMARKS AND HYPOTHESIS

Stress might be considered as a condition where IS, CNS, and ES work together: such network is achieved by cortisol, norepinephrine, melatonin, and cytokines, with particular emphasis on interleukin‐1β.

**FIGURE 3:**
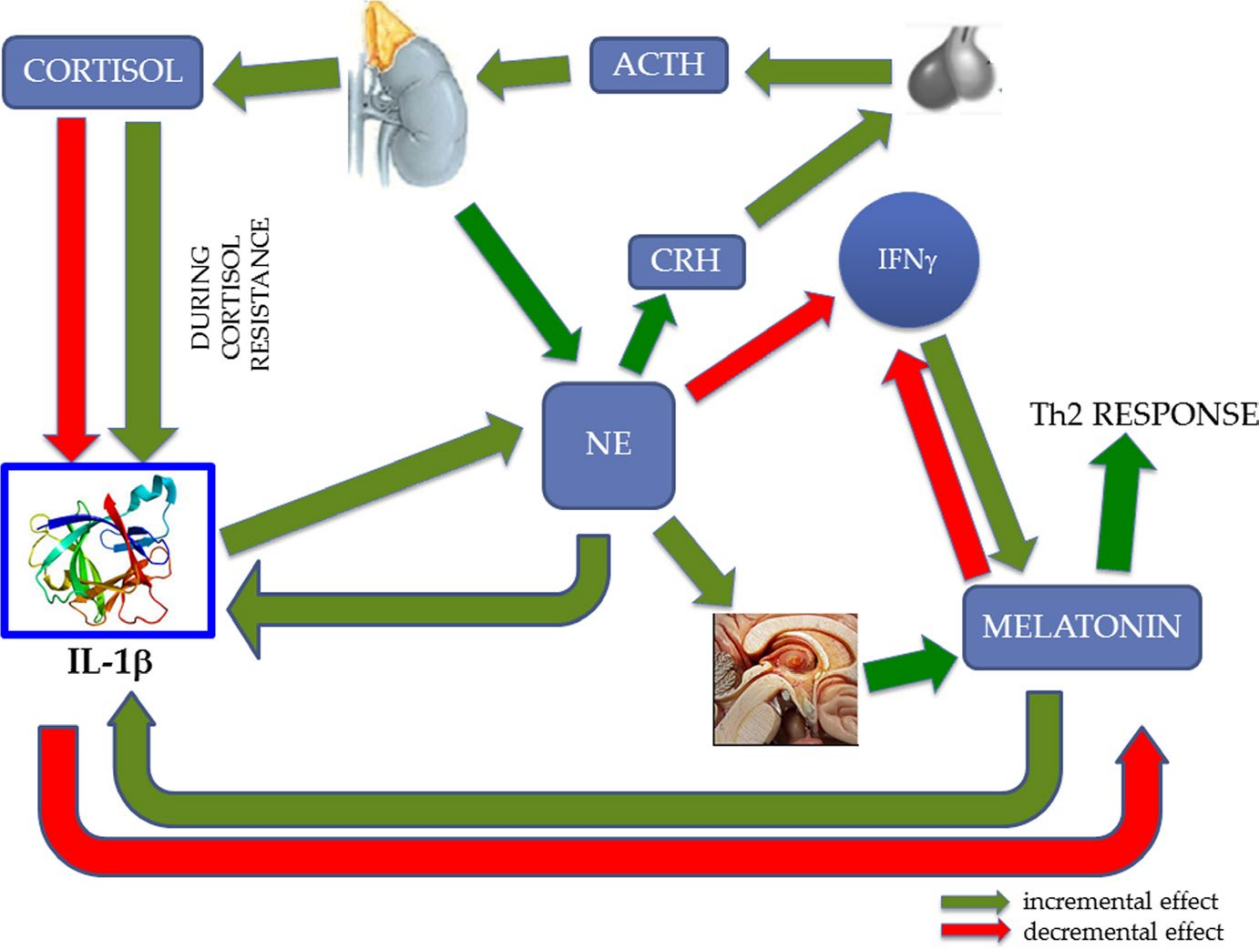
Interplay between central nervous system, endocrine system, and immune system. Under physiological conditions, norepinephrine (NE) activates the HPA axis and determines the production of cortisol that in turn downregulates the production of IL‐1β. During chronic stress and especially glucocorticoid resistance state, the increment of IL‐1β transforms this inflammatory signal in nervous signal (NE). In turn, NE uses the ES (melatonin and cortisol) to counterbalance again IL‐1β, but this control cannot function during cortisol resistance. NE induces eventually a skewing in the immune response, with reduction in IFN‐γ and Th1 activation, and with unbalancing toward Th2 response. The protein structure of IL‐1β was taken from the RCSB‐protein data bank (www.rcsb.org/)

Based on the above‐cited literature, we would like to propose a hypothesis able to correlate these different signaling paracrine and hormones. Despite the difficulty to study these systems in their interactions with each other, an important control that every substance reciprocally exerts on each other can be observed and a complex interplay between them can be proposed (Figure 3). Accordingly, NE determines an incremental effect on melatonin and CRH, with the latter able to increment cortisol; NE is also capable to reduce IFN‐γ, thus NE might be considered an anti‐Th1 effector. Moreover, this effect on Th1 would be incremented by melatonin that acts as pro‐Th2. Also cortisol was demonstrated to increase the Th2 response, and melatonin, like NE, can reduce IFN‐γ.

Considering cortisol, we may observe that it determines a decrement in IL‐1β and an increment in NE, then it would generate an anti‐inflammatory response. Considering the role of IL‐1β we can observe that it increments NE, reduces melatonin, but because the latter increases IL‐1β it might play as a negative feedback on this cytokine. We know that IL‐1β produced by glial cell, neurons, and IS, is able to pass through BBB determining Sickness Behaviour Symptoms. Moreover, Palumbo et al. (Palumbo et al., 2010) gave evidence that strains of mice had different resistance to the stress and the strain less resistant showed an important IL‐1 increment following aggression. Important is the observation (Ashley et al., 2017) that demonstrated IL‐1 was able to stimulate HPA axis through increment of NE in brain areas rich in CRH neurons and an inhibition of HPG axis through decrement of NE in the brain areas rich in GnRH neurons (Figure 2).

Based on these considerations, we may hypothesize IL‐1β to be the main cause of adverse stress effects and also the main biomarker of this effects, because it determines an increment of NE that induces a cascade effects on melatonin and cortisol that in turn act on IS and also on IL‐1β itself; particularly important appears the cortisol effect during cortisol resistance where cortisol increases IL‐1β, instead to reduce it. High levels of GC in chronic stress (i.e., in depression) cause resistance to glucocorticoid feedback on the HPA axis, and this developed glucocorticoid resistance allows the escape of proinflammatory signaling pathways from normal feedback inhibition (Pariante, 2017). Then, we might summarize all observations with this hypothesis: During chronic stress, the increment of IL‐1β transforms this inflammatory signal in nervous signal (NE), in turn, this uses the ES (melatonin and cortisol) to counterbalance again IL‐1β, but this control could not function during cortisol resistance as in physiological circumstances. A vicious loop ensues that increments all mediators, unbalancing IS, ES, and CNS network. This increase in IL‐1β would appear to occur above all when the individual instead to cope the stressful event, succumbs to it showing the Sickness Behaviour Symptoms. These symptoms derive from IL‐1β, then they could be the outcome of this loop, particularly the increment of IL‐1β might not be counteracted by cortisol and this event would be able to unbalance CNS, IS, ES, network polarizing IS toward a Th2 response. These effects could represent the link between stress and autoimmunity and carcinogenesis through a reduction of control mechanism on tumor growth feature of Th1 response.

Considering all said above it might appear useful measuring IL‐1β to evaluate stress and stress effects; it could be proposed as an important benchmark to evaluate stress jointly with cortisol and melatonin, especially when it is a response at events perceived as aggressive.

## CONFLICT OF INTEREST

All authors have no conflict of interest to declare.

## AUTHOR CONTRIBUTION

Roberto Zefferino conceived this review. Roberto Zefferino, Massimo Conese, and Sante Di Gioia searched literature. Roberto Zefferino, Massimo Conese, and Sante Di Gioia draw the figures. Roberto Zefferino and Massimo Conese wrote the manuscript. Sante Di Gioia edited the manuscript. All authors approved the final manuscript as submitted.

### Peer Review

The peer review history for this article is available at https://publons.com/publon/10.1002/brb3.1960.
